# Distribution and predictors associated with the use of breast cancer screening services among women in 14 low-resource countries

**DOI:** 10.1186/s12889-020-09557-w

**Published:** 2020-09-29

**Authors:** Rashidul Alam Mahumud*, Jeff Gow, Syed Afroz Keramat, Sonja March, Jeff Dunn, Khorshed Alam, Andre M. N. Renzaho

**Affiliations:** 1 School of Social Sciences, Western Sydney University, Penrith, New South Wales 2751 Australia; 2 Translational Health Research Institute (THRI), Western Sydney University, Sydney, New South Wales Australia; 3 Health Economics and Policy Research, School of Commerce, Centre for Health Research, University of Southern Queensland, Toowoomba, Queensland 4350 Australia; 4 School of Accounting, Economics and Finance, University of KwaZulu-Natal, Durban, 4000 South Africa; 5 Department of Economics, American International University-Bangladesh, Dhaka, 1212 Bangladesh; 6 School of Psychology and Counselling, University of Southern Queensland, Toowoomba, Queensland 4300 Australia; 7 Cancer Research Centre, Cancer Council Queensland, Fortitude Valley, Brisbane, QLD 4006 Australia; 8 Prostate Cancer Research Foundation of Australia, St Leonards, New South Wales 2065, 40 Australia

**Keywords:** Breast cancer screening services, Low-resource countries, Reproductive women, Determinants

## Abstract

**Background:**

Breast cancer is one of the leading public health problem globally, especially in low-resource countries (LRCs). Breast cancer screening (BCS) services are an effective strategy for early determining of breast cancer. Hence, it is imperative to understand the utilisation of BCS services and their correlated predictors in LRCs. This study aims to determine the distribution of predictors that significantly influence the utilisation of BCS services among women in LRCs.

**Methods:**

The present study used data on 140,974 women aged 40 years or over from 14 LRCs. The data came from country Demographic and Health Surveys (DHS) between 2008 and 2016. Multivariate logistic regression analysis was employed to investigate the significant predictors that influence the use of BCS services.

**Results:**

The utilisation of BCS services was 15.41%, varying from 81.10% (95% CI: 76.85–84.73%) in one European country, to 18.61% (95% CI: 18.16 to 19.06%) in Asian countries, 14.30% (95% CI: 13.67–14.96%) in American countries, and 14.29% (95% CI: 13.87–14.74%). Factors that were significantly associated to increase the use of BCS services include a higher level of education (OR = 2.48), advanced age at first birth (> 25 years) (OR = 1.65), female-headed households (OR = 1.65), access to mass media communication (OR = 1.84), health insurance coverage (OR = 1.09), urban residence (OR = 1.20) and highest socio-economic status (OR = 2.01). However, obese women shown a significantly 11% (OR = 0.89) lower use of BSC services compared to health weight women.

**Conclusion:**

The utilisation of BCS services is low in many LRCs. The findings of this study will assist policymakers in identifying the factors that influence the use of BCS services. To increase the national BCS rate, more attention should be essential to under-represented clusters; in particular women who have a poor socioeconomic clusters, live in a rural community, have limited access to mass media communication, and are have a low level educational background. These factors highlight the necessity for a new country-specific emphasis of promotional campaigns, health education, and policy targeting these underrepresented groups in LRCs.

## Background

Breast cancer (BC) is one of the public health problem worldwide and the second leading cause of overall death due to cancer [1]. In 2018, nearly 2.1 million women experienced BC and 627,000 women died from it (i.e., accounting for fiften percent of all cancer deaths) [2]. The rate of incidence, mortality and survival of BC vary across countries [3, 4]. The incidence rate of BC is increasing in low-resource countries (LRCs) due to demographic transition, changing disease patterns, unhealthy lifestyles, and behaviors that lead to a high risk of BC incidence [5–9]. Although the incidence rate of BC in developed countries is 89 per 100,000 women, it is below 40 in LRCs [10]. However, in recent times, this low incidence rate in LRCs have been increasing at a faster rate compared to developed countries [11]. The mortality rate of BC is also increasing in this setting; for instance, about 60% of women die due to BC in LRCs [10]. The five-year survival rates of BC varies to a great extent globally, ranging from 80% (in developed countries) to less than 40% (in developing countries) [12]. Therefore, the burden of BC is particularly underdetermined in LRCs.

Breast cancer disproportionately affects more among reproductive women (aged 15–49 years) in LRCs (23% of new cases) than developed countries (10% of new cases) [13]. The recommended starting age for routine BCS varies widely as well due to lack of government recommendations [14–19]. However, a few proportion (2.2%) of aged women (i.e., aged 40–69 years) had utilised screening services in LRCs [20].

In LRCs, some predictors that could decline the efficacy of BCS services include a younger women with the lower incidence of BC, poor health status, and a prevalence of biologically destructive sub-types for which patient outcomes are associated with lower utilisation of screening services [13]. Conversely, BCS could have a greater impact in LRCs if it promotes BC awareness, knowledge, percention, and early screning of symptomatic disease. For instance, there may be greater effects than would be anticipated in developed settings, where strong health systems and higher levels of awareness reduce the opptornuty of BCS primarly to the detection of asymptomatic disease. Also, LRCs recurrently lack the requisite skeletal structure to corroborate high-quality mammography and afterwards healthcare services [14], which in commit may be restricted by a lack of resources (i.e., x-ray films, and mammography) [15, 16]. In LRCs, most monographs are provided through private hospitals, making it unaffordable [17, 18]. In this context, evidence is required to lead large-scale BCS in LRCs considering socio-economic status and cultural affiliations, as effects on BC-specific death rates remain unclear. Inadequate cancer registration, course of treatment, and diagnosis throughout LRCs also limit the influence on screening services as well as their evaluation, and this must therefore be strengthened simultaneously.

Regular screening is an effective way of detecting BC [11]. Furthermore, the risk of BC-related mortality rates is significantly lower among women in developed countries who had experienced with BCS [19]. Despite the benefits of screening, the utilisation of BCS services are relatively low in LRCs, compared to high income countries (HICs) [11, 12]. Factors that can influence participation in BCS services vary in different countries’ settings [20]. Some studies have found that socio-economic factors (such as age distribution, marital status, socioeconomics group) are the leading driving force behind utilising BCS services in LRCs [11, 21, 22]. Apart from socio-demographic and economic factors, access to health care services, and health insurance coverage are significantly correlated with higher utilisation of BCS services [23, 24]. In addition, screening behaviors [25], prior knowledge [26], and lack of access to a physician [27] are more likely to influence women’s participation in BCS services. Religion, cultural beliefs, social barriers, and ethnicity related factors are also the leading factors responsible for the lower use of BCS services [28, 29].

Some limitations have been observed in previous research that has focused on the determinants of BCS services. The most common limitations are small populations and/or limited study settings (e.g., targeting only a particular region in a country). Central policy-making that has aimed to prevent the burden of BC based on the outcomes of small study settings is problematic across countries, and this is part of an ongoing debate about breast cancer-related research [30]. Therefore, it is important to conduct large-scale studies the findings of which can offer generalisations of the use of BCS services among women. To provide national efforts to decline the incidence of women’s cancers (e.g., breast cancer), studies required to generate evidence in terms of specific and estimable information about adequate cancer screening services.

The present study analysed data from 140,974 reproductive women and living in 14 LRCs to examine the current distribution of BCS use and identify potential factors that influence screening use. The findings may offer understanding for evidential priority health interventions across the ongoing country-specific health system. Additionally, significant findings are deliberated considering national health policy in these low-resource countries. This study aimed to indentify the distribution of utilisation of BCS services and to investigate the predictors that have a significant influence on BCS services among women in low-resource settings.

## Methods

### Study design

The design of the study was cross sectional, using the latest Demographic and Health Survey (DHS) data. Data were generated from the latest DHS, including 14 LRCs from 2008 to 2016 [31–44]. The present study was a sub-study, which was generated from the latest DHS survey. Health, demographic, and health care services associated data were captured in this survey, in the context of mostly LRCs. The details of the survey were explained elsewhere [31–44].

### Sampling and sample size calculation

In DHS surveys, a two-stage cluster sampling was occupied [45]. During the *first stage*, *primary sampling units* (PSUs) were drawn from a frame respondent list with probability proportional to a size measure. A PSU was commonly a geographically area, named an enumeration area (EA), including a number of households that were made from the recent population census. A number of households were chosen from a list of households pointedly as part of an introducing technique in the selected PSUs in the second stage. The required sample size was estimated and explained elsewhere [45], using the following three equations

1 $$ {n}_{opt}=\frac{C}{c_1+{c}_2{m}_{opt}\ } $$

2 $$ C={c}_1n+{c}_2 nm $$

3 $$ {m}_{opt}=\sqrt{\frac{\left(1-\rho \right){c}_1}{\rho {c}_2}} $$

where, *n*_*opt*_ was denoted estimated sample, *C* was defined the aggregated cost of the survey, *c*_1_ and *c*_2_ were explained the unit cost per interview and the unit cost per interview, respectively. *n* was denoted the total amount of PSUs, *m* was the number of respondents in each PSU, and *ρ* was defined the intracluster correlation.

### Data collection procedure

**Fig. 1:**
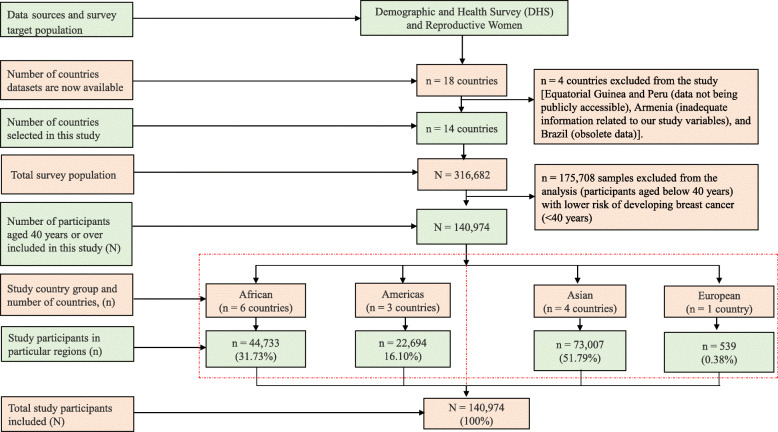
Distribution of study sample

Data were collected from target participants. The target participants were the reproductive women (e.g., 15 to 49 years’ group). Participants were surveyed using the DHS survey instruments. Quantitative structural questionnaires were used to collect data by Measure DHS retrospectively. The total number of study participants was 140,974 reproductive women living in 14 LRCs (Fig. 1).

### Study settings

The disease, screening knowledge, practices, or outcomes related questions were asked across all 14 LRCs. The DHS surveys have been implemented across 90 countries, breast cancer screening questions occurred in only 18 of them. Breast cancer questions (have been added into relatively few DHS surveys, but their absolute inclusion has increased since 1984. Of these countries, data on the utilisation of BCS services were considered from 14 LRCs [31–44]: Albania (survey years: 2008–09), Burkina Faso (survey year: 2010), Colombia (survey year: 2015), Ivory Coast (survey years: 2011–12), Dominican Republic (survey year: 2013), Egypt (survey year: 2015), Honduras (survey years: 2011–12), India (survey years: 2015–16), Jordan (survey year: 2012), Kenya (survey year: 2015), Lesotho (survey year: 2014), Namibia (survey year: 2013), Philippines (survey year: 2013), and Tajikistan (survey year: 2012) (Table 1). However, the four countries (e.g., Equatorial Guinea, Peru, Armenia, and Brazil) were excluded from this study due to their data not being publicly accessible, inadequate information related to our study variables, and obsolete data, so in each case the data were incomplete. Study countries that were distributed across four international geographical lens based on availability of survey data (Fig. 1).

**Table 1:** Distribution of study sample

Study country	Survey year	Number of participants	The utilisation of BCS services	95% confidence interval (CI)	
		weighted sample, (n)	weighted percentage, (%)	Low bound	Upper bound
Albania	2008–09	539	80.82	77.28	83.93
Burkina Faso	2010	473	10.56	8.09	13.68
Colombia	2015	3075	25.26	23.76	26.83
Cote d’Ivoire	2011–12	5382	5.27	4.70	5.90
Dominican Republic	2013	6643	10.28	9.57	11.03
Egypt	2015	16,973	1.84	1.64	2.05
Honduras	2011–12	12,975	20.36	19.67	21.06
India	2015–16	43,502	5.63	5.41	5.85
Jordan	2012	18,255	36.67	35.98	37.37
Kenya	2015	11,847	24.61	23.85	25.40
Lesotho	2014	3993	8.47	7.64	9.37
Namibia	2013	6065	37.80	36.59	39.03
Phipillines	2013	9384	7.08	6.58	7.62
Tajikistan	2012	1866	63.70	61.49	65.85
**Overall**	2008–16	140,974	15.41	15.22	15.60

### Description of study variables

#### Dependent variables

The use of BCS services served as the dependent variable. As part of data collection on BCS services, the participants were asked questions by the DHS trained surveyor [31–44]. The dependent variable (i.e., the ever use of BCS practice) was restricted to DHS data, which was of a quantitative cross-sectional nature, based on the participants’ self-reported responses. For a particular study country, only one question related to the ever utilisation of breast cancer screening services (or if they had ever had a breast examination) was identified in the DHS datasets [46]. According to the DHS guideline, the dependent variable was expressed as a dichotomous response (‘yes’ if the participants had ever used a breast cancer screening service or had ever had a breast examination or ‘no’ otherwise).

#### Explanatory variables

Explanatory variables were selected from the available data sources that were validated based on published research articles on breast cancer screening, epidemiological studies [23, 47–57]. Different explanatory variables were selected for each factor. The predisposing factors were considered based on participant’s age, education, household head, the age at first birth, and parity. All the predictors under predisposing factors were categorical variables. Age was categorised into two groups: 40 to 44 years or ≥ 45 years at high risk of occurring BC. Education was classified as illiterate, primary education, secondary education, or higher education. The gender of the household head was defined as male-headed or female-headed. The age of the respondents at first birth was distributed into four groups: < 18 years, 18–20 years, 21–25 years, and > 25 years old. The number of childbirths were recoded into three groups: < 4 births, 4–5 births, and > 5 births. Household access to mass media coverage and the status of health insurance coverage served as proxies of enabling factors. Media coverage was denoted as ‘yes’ or ‘no’. The status of health insurance was dichotomous: ‘yes’ if the household was insured, and ‘no’ if uninsured. Another factor, the condition of participants’ body mass index (BMI) was categorised into three groups: underweight, healthy weight, and overweight. BMI was used to measure the participants’ weight status in the following way: underweight (≤ 18.5 kg/m^2^), healthy weight (18.51–24.99 kg/m^2^), overweight (25–29.99 kg/m^2^), and obese (≥ 30.00 kg/m^2^). Another important control variable, the participant’s residence, was classified as urban or rural, and these factors were dichotomous. In the context of LRCs, urban residence covers cities and towns while rural residence includes villages and hamlets. DHS has developed a wealth index using ownership of durable assets [58], which has demonstrated sound psychometric properties [59]. This wealth index variable served as another predictor in the model. The wealth index was classified such as poorest (Q_1_; 20% lowest), poorer (Q_2_), middle (Q_3_), richer (Q_4_), and richest (Q_5_; 20% highest).

### Data analysis

In the descriptive analyses, the participant’s characteristics were presented using frequencies (n) and percentages (%). The category level found to be at the lower risk for using BCS services was considered as the reference catagory to construct unadjusted and adjusted odds ratios (ORs) using multivariate logistic regression analysis, with a 95% CI. A series of diagnostics were tested in the analytical exploration. For instance, the Hosmer and Lemeshow statistic used to execute the goodness of fit test for model [60]. The variance inflation factor (VIF) was also used to detect if any multicollinearity existed among explanatory variables in the model [61]. The ROC (receiver operating characteristic) curve was used to ensure the best predictive power of the fitted model [62]. The sampling weight was adjusted in the analyses, which was derived from the DHS data [63]. Data analysis was performed using Stata/SE 13. A *p*-value of 0.05 or below was considered a significant level in this study.

## Results

### Characteristics of the participants

Approximately 47.25% of the total participants were aged 40–44 years, whereas 52.75% were aged 45 years or more (Table 2). Fifty-nine percent of all participants had ‘no formal or only primary level educational background’, combined, whereas approximately only 10% of participants had completed higher education. A high proportion of participants had no formal education in Asian countries (~ 36%), followed by 29% in African countries. Furthermore, the overall illiteracy rate was found to be only 29.08%. Approximately two-third of households were male-headed, a high proportion was observed in the European country (97.63%), followed by Asian countries (85.9%), and African countries (71.97%). Approximately 67% of women had delivered four children or more. Only 22.61% of participants had insured, and 59% of women were overweight (26%) or obese (31%). Survey results also revealed that nearly 55% of women lived in a rural community, with the highest proportion found in African countries (65%), followed by 59% in the European country, 55% in Asian countries, and the lowest in Americas countries (37%). About 38% of respondents were from a low socio-economic status background.

**Table 2:** Background characteristics of study participants

Participants characteristics	International geographical regions				Full sample, n (%)
	Americas, n (%)	Asian, n (%)	African, n (%)	European, n (%)	
Age in years					
40–44 years	11,401 (50.24)	31,468 (43.1)	23,344 (52.19)	395 (73.25)	66,608 (47.25)
≥ 45 years	11,293 (49.76)	41,539 (56.9)	21,388 (47.81)	144 (26.75)	74,365 (52.75)
Educational level					
*No education*	1519 (6.70)	26,552 (36.37)	12,916 (28.87)	3 (0.57)	40,990 (29.08)
*Primary*	13,289 (58.56)	12,983 (17.78)	15,739 (35.19)	279 (51.79)	42,290 (30.00)
*Secondary*	5348 (23.56)	24,838 (34.02)	13,299 (29.73)	215 (39.92)	43,699 (31.00)
*Higher*	2538 (11.18)	8629 (11.82)	2779 (6.21)	42 (7.73)	13,988 (9.92)
Head of the household					
*Male*	14,876 (65.55)	62,716 (85.9)	32,195 (71.97)	527 (97.63)	11,0314 (78.25)
*Female*	7817 (34.45)	10,292 (14.1)	12,538 (28.03)	13 (2.37)	30,660 (21.75)
Respondent’s age at 1st birth					
*< 18 years*	6389 (28.28)	16,940 (26.65)	11,047 (24.7)	3 (0.56)	34,379 (26.16)
*18–20 years*	7776 (34.42)	21,212 (33.37)	15,178 (33.93)	75 (14.01)	44,241 (33.66)
*21–25 years*	5908 (26.15)	19,990 (31.44)	13,845 (30.95)	320 (60.00)	40,064 (30.48)
*> 25 years*	2520 (11.15)	5430 (8.54)	4660 (10.42)	136 (25.42)	12,747 (9.7)
Number of childbirths					
*< 4*	8739 (38.51)	23,718 (37.28)	9943 (22.23)	428 (79.32)	42,828 (32.55)
*4–5*	7082 (31.21)	21,125 (33.2)	15,056 (33.66)	98 (18.24)	43,362 (32.95)
*> 5*	6873 (30.28)	18,780 (29.52)	19,734 (44.11)	13 (2.44)	45,400 (34.50)
Mass media exposure					
*No*	1376 (6.06)	13,856 (18.98)	8654 (19.35)	9 (1.61)	23,895 (16.95)
*Yes*	21,318 (93.94)	59,151 (81.02)	36,079 (80.65)	531 (98.39)	11,7079 (83.05)
Health insurance coverage					
*No*	13,704 (69.88)	30,809 (70.82)	38,953 (87.09)	409 (75.78)	83,874 (77.39)
*Yes*	5906 (30.12)	12,694 (29.18)	5773 (12.91)	131 (24.22)	24,504 (22.61)
Body mass index					
*Under weight*	230 (1.18)	4980 (8.92)	1401 (3.88)	4 (0.73)	6615 (5.91)
*Normal weight*	4646 (23.82)	22,948 (41.12)	10,075 (27.93)	208 (38.56)	37,877 (33.84)
*Overweight*	6898 (35.37)	15,801 (28.31)	9890 (27.42)	225 (41.71)	32,814 (29.32)
*Obese*	7728 (39.62)	12,082 (21.65)	14,709 (40.77)	103 (19.01)	34,621 (30.93)
Community					
*Urban*	14,376 (63.35)	33,073 (45.3)	15,612 (34.90)	222 (41.19)	63,283 (44.89)
*Rural*	8318 (36.65)	39,934 (54.7)	29,121 (65.10)	317 (58.81)	77,690 (55.11)
Wealth quintile					
*Q*_*1*_ *(Poorest 20%)*	3552 (15.65)	11,835 (16.21)	9690 (21.66)	98 (18.14)	25,175 (17.86)
*Q*_*2*_	4135 (18.22)	14,552 (19.93)	9253 (20.69)	108 (20.10)	28,048 (19.90)
*Q*_*3*_	5265 (23.2)	15,321 (20.99)	8821 (19.72)	131 (24.22)	29,538 (20.95)
*Q*_*4*_	4877 (21.49)	15,884 (21.76)	8388 (18.75)	104 (19.24)	29,253 (20.75)
*Q*_*5*_ *(Richest 20%)*	4866 (21.44)	15,415 (21.11)	8580 (19.18)	99 (18.30)	28,960 (20.54)
*Total observations*	22,694 (16.10)	73,007 (51.79)	44,733 (31.73)	539 (0.38)	140,974 (100)

All estimates were sampling weight adjusted

### The utilisation of BCS services across geographical areas

The overall utilisation of BCS services was 15.41% (Table 3), whereas the utilisation rate was comparatively higher among participants aged 40 to 44 years (16.43%), compared to participants aged 45 years or over (14.49%). The utilisation of BCS services varied across reagions, for instance, 81.10% in the European country, 18.61% in Asian countries, 14.30% in American countries, and 14.29% in African countries. Several countries had a lower utilisation rate of BCS services. For example, the screening participation rate was less than 11% in Burkina Faso, the Dominican Republic, Egypt, India, Lesotho, and in the Philippines. The utilisation of screening services increased with higher levels of education among participants, for both the following age group: 40 to 44 years and ≥ 45 years old. For instance, overall only 6% of women (i.e., 6.73% of women aged 40 to 44 years and 5.78% of women aged 45 years or over) utilised screening services who had no formal education, whereas 29% of higher educated women (i.e., 32.97% of women aged 40–44 years and 27.27% of women aged 45 years or over) utilised BCS services. The use of BCS services among women aged 40 to 44 years from female-headed households (18.38%) was slightly higher compared with the use of BCS services among women aged 45 years or over (16.36%). Furthermore, approximately 16% of women from insured households aged 40–44 years used BCS services. Among participants (≥ 45 years old or over), with the highest proportion observed in American countries (49.46%), followed by 30.97% in African countries and the lowest in Asian countries (2.87%). Similarly, the use of screening services among women was very low irrespective of BMI status (e.g., 8.43% for underweight, 11.85% for a healthy weight, 16% for overweight or obese). Regarding geographic location, the higher proportion of participants who lived in urban communities participated in BCS services compared to participants who lived in a rural community. In addition, the overall use of BCS services was found to be highest in the wealthiest socio-economic status households (22%), followed by middle-class households (16%) and the poorest households (12%), respectively.

**Table 3:** Distribution of the utilisation of BCS services across geographical regions

Participants characteristics	The utilisation of BCS services, (%)								Overall	
	Americas		Asian		African		European			
	40–44 years	≥ 45 years	40–44 years	≥ 45 years	40–44 years	≥ 45 years	40–44 years	≥ 45 years	40–44 years	≥ 45 years
Educational level										
*No education*	5.66	11.58	7.83	6.55	4.89	3.12	0.00	100	6.73	5.78
*Primary*	13.21	17.28	7.35	8.86	16.14	16.29	71.73	76.28	13.63	14.05
*Secondary*	17.81	30.12	23.85	18.71	17.89	18.57	88.30	88.73	21.53	20.22
*Higher*	19.38	34.92	39.73	26.01	26.30	24.69	98.00	100	32.97	27.27
Head of the household										
*Male*	14.42	22.78	17.76	13.50	12.21	10.10	79.48	84.08	15.92	13.95
*Female*	15.48	18.99	18.06	10.23	20.20	20.92	100.00	33.25	18.38	16.36
Respondent’s age at 1st birth										
*< 18 years*	12.10	15.74	9.23	8.08	12.17	12.20	40.96	100	10.74	10.79
*18–20 years*	13.65	20.39	18.48	12.95	15.17	13.11	78.71	78.02	16.57	14.30
*21–25 years*	17.07	24.46	23.54	18.44	13.90	14.10	79.61	82.94	19.71	18.15
*> 25 years*	19.79	29.57	30.12	21.83	17.78	14.19	82.52	83.07	23.78	21.26
Number of childbirths										
*< 4*	18.87	29.09	9.93	7.45	18.39	18.84	81.40	87.02	15.21	14.10
*4–5*	14.67	19.61	21.41	13.70	13.91	12.66	77.72	64.59	17.71	14.35
*> 5*	7.93	15.50	25.51	23.86	12.26	11.47	57.25	100	17.23	17.24
Mass media exposure										
*No*	11.21	12.08	8.77	6.73	12.34	11.08	28.67	100	10.41	8.40
*Yes*	14.99	22.03	19.92	14.45	14.85	13.79	80.92	82.90	17.68	15.72
Health insurance coverage										
*No*	13.68	20.30	7.33	6.03	11.58	10.75	75.98	81.06	11.03	10.51
*Yes*	16.69	17.06	3.90	2.87	33.76	29.74	92.05	90.28	15.71	12.07
Body mass index										
*Under weight*	15.90	12.87	6.80	6.37	14.95	13.12	0.00	0.00	8.99	7.90
*Normal weight*	12.47	12.64	11.16	7.83	15.79	16.16	80.24	71.89	13.32	10.51
*Overweight*	15.58	20.78	20.82	10.14	15.82	13.56	80.26	96.95	18.73	13.60
*Obese*	14.98	22.15	21.87	24.52	9.42	8.26	81.80	82.20	15.18	17.29
Community										
*Urban*	16.87	25.51	25.08	19.00	19.18	17.12	89.95	88.56	21.91	20.16
*Rural*	11.19	14.18	11.00	8.45	11.70	11.29	73.74	77.99	11.74	10.08
Wealth quintile										
*Q*_*1*_ *(Poorest 20%)*	7.79	12.19	17.86	13.80	8.03	5.81	67.04	74.21	12.84	10.71
*Q*_*2*_	12.77	16.81	14.65	10.88	12.59	9.68	72.48	58.81	14.00	11.45
*Q*_*3*_	10.97	14.15	16.76	12.01	12.23	14.63	79.59	84.23	14.56	13.28
_*Q4*_	14.84	22.63	17.76	13.54	17.44	14.32	83.26	95.93	17.42	15.31
*Q*_*5*_ *(Richest 20%)*	25.37	39.11	22.06	14.71	22.55	23.01	99.16	87.54	23.30	20.72
**Total participants, %**	14.77	21.41	17.79	12.99	14.34	13.30	79.99	83.09	16.43	14.49
Overall, % (95% CI)	14.30 (13.67, 14.96)		18.61 (18.16, 19.06)		14.29 (13.87, 14.74)		81.10 (76.85, 84.73)		15.41 (15.22–15.60)	

All estimates were sampling weight adjusted. The percentage was presented by row wise

### Factors influencing of the use of BCS services

Predisposing factors, such as education, age at first birth, and female-headed households, showed a significant positive association with higher use of BCS services after controlling other factors (Table 4). The increased level of education of the participants significantly influenced the higher use of BCS services. Higher educated participants were more likely to utilise BCS services (OR = 2.48, 95% CI: 2.25–2.73) compared to participants with no formal education. Similar associations were found in African countries (OR = 3.65, 95% CI: 3.08–4.32), and in the European country (OR = 3.24, 95% CI: 2.83–3.73). The study also exhibited that a higher age at first birth was associated with the utilisation of BCS services (OR = 1.65, 95% CI: 1.52–1.78). Regarding the head of household, participants from female-headed households were 1.65 times more likely to utilise BCS services (OR = 1.65, 95% CI: 1.58–1.73) compared to participants from male-headed households.

**Table 4:** Factors influencing the utilisation of BCS services by geographical regions

Participants characteristics	Americas		Asian		African	
	Un-adjusted OR (95% CI)	Adjusted OR (95% CI)	Un-adjusted OR (95% CI)	Adjusted OR (95% CI)	Un-adjusted OR (95% CI)	Adjusted OR (95% CI)
Age group						
40–44 years (= ref)	1.00	1.00	1.00	1.00	1.00	1.00
≥ 45 years	1.57 (1.47, 1.68)	1.48 (1.37, 1.60)	0.69 (0.66, 0.72)	0.76 (0.70, 0.82)	0.92 (0.87, 0.97)	0.91 (0.85, 0.98)
Educational level						
*No education (= ref)*	1.00	1.00	1.00	1.00	1.00	1.00
*Primary*	1.71 (1.43, 2.04)	1.48 (1.22, 1.80)	1.19 (1.10, 1.29)	0.67 (0.58, 0.76)	4.68 (4.24, 5.16)	3.82 (3.39, 4.31)
*Secondary*	2.92 (2.43, 3.50)	1.67 (1.35, 2.07)	3.54 (3.34, 3.74)	0.83 (0.74, 0.94)	5.38 (4.87, 5.93)	3.47 (3.05, 3.94)
*Higher*	3.41 (2.81, 4.14)	1.12 (0.88, 1.43)	6.23 (5.84, 6.65)	1.10 (0.84, 1.44)	8.29 (7.33, 9.37)	3.65 (3.08, 4.32)
Gender of the household head						
*Male (= ref)*	1.00	1.00	1.00	1.00	1.00	1.00
*Female*	0.92 (0.86, 0.99)	0.99 (0.91, 1.07)	0.83 (0.78, 0.89)	1.43 (1.29, 1.59)	2.05 (1.94, 2.16)	1.81 (1.69, 1.95)
Respondent’s age at 1st birth						
*< 18 years (= ref)*	1.00	1.00	1.00	1.00	1.00	1.00
*18–20 years*	1.25 (1.14, 1.37)	1.15 (1.04, 1.27)	1.96 (1.83, 2.09)	1.12 (1.01, 1.25)	1.19 (1.11, 1.28)	0.99 (0.90, 1.08)
*21–25 years*	1.62 (1.47, 1.78)	1.28 (1.15, 1.44)	2.78 (2.61, 2.97)	1.20 (1.08, 1.34)	1.17 (1.09, 1.26)	0.79 (0.71, 0.87)
*> 25 years*	2.03 (1.81, 2.28)	1.40 (1.20, 1.64)	3.60 (3.32, 3.9)	1.80 (1.51, 2.14)	1.38 (1.25, 1.52)	0.74 (0.64, 0.84)
Number of childbirths						
*< 4 (= ref)*	1.00	1.00	1.00	1.00	1.00	1.00
*4–5*	0.67 (0.62, 0.73)	0.91 (0.82, 1.00)	2.24 (2.12, 2.38)	1.32 (1.20, 1.46)	0.67 (0.63, 0.72)	0.95 (0.87, 1.04)
*> 5*	0.46 (0.42, 0.50)	0.87 (0.77, 0.98)	3.49 (3.30, 3.69)	1.53 (1.35, 1.73)	0.59 (0.55, 0.63)	0.99 (0.89, 1.09)
Mass media exposure						
*No (= ref)*	1.00	1.00	1.00	1.00	1.00	1.00
*Yes*	1.72 (1.45, 2.03)	0.99 (0.83, 1.19)	2.45 (2.29, 2.62)	0.81 (0.72, 0.91)	1.25 (1.17, 1.35)	0.92 (0.84, 1.01)
Health insurance coverage						
*No (= ref)*	1.00	–	1.00	1.00	1.00	1.00
*Yes*	0.99 (0.91, 1.08)		0.48 (0.44, 0.54)	0.53 (0.47, 0.59)	3.70 (3.47, 3.94)	2.25 (2.07, 2.45)
Body mass index						
*Under weight*	1.15 (0.79, 1.68)	1.48 (1.00, 2.19)	0.69 (0.61, 0.77)	0.84 (0.73, 0.96)	0.86 (0.74, 1.01)	1.17 (0.99, 1.38)
*Normal weight (= ref)*	1.00	1.00	1.00	1.00	1.00	1.00
*Overweight*	1.55 (1.39, 1.72)	1.42 (1.28, 1.59)	1.74 (1.63, 1.85)	1.03 (0.93, 1.15)	0.91 (0.84, 0.98)	0.80 (0.73, 0.87)
*Obese*	1.59 (1.43, 1.76)	1.41 (1.26, 1.57)	2.96 (2.78, 3.14)	1.11 (0.96, 1.28)	0.51 (0.47, 0.55)	0.46 (0.42, 0.51)
Community						
*Urban*	1.86 (1.72, 2.00)	1.22 (1.11, 1.35)	2.66 (2.55, 2.77)	1.21 (1.08, 1.34)	1.71 (1.62, 1.81)	0.94 (0.86, 1.03)
*Rural (= ref)*	1.00	1.00	1.00	1.00	1.00	1.00
Wealth quintile						
*Q1 (Poorest 20%) (= ref)*	1.00	1.00	1.00	1.00	1.00	1.00
*Q2*	1.57 (1.37, 1.80)	1.52 (1.29, 1.79)	0.76 (0.71, 0.82)	0.82 (0.72, 0.94)	1.69 (1.52, 1.87)	1.63 (1.44, 1.85)
*Q3*	1.29 (1.12, 1.48)	1.11 (0.94, 1.32)	0.87 (0.81, 0.93)	0.82 (0.70, 0.96)	2.07 (1.87, 2.28)	1.63 (1.44, 1.85)
*Q4*	2.05 (1.80, 2.34)	1.64 (1.38, 1.95)	0.96 (0.90, 1.02)	0.85 (0.71, 1.02)	2.54 (2.30, 2.80)	2.49 (2.18, 2.85)
*Q5 (Richest 20%)*	4.26 (3.76, 4.82)	3.39 (2.84, 4.06)	1.15 (1.07, 1.22)	0.83 (0.68, 1.01)	3.94 (3.59, 4.32)	3.58 (3.08, 4.17)
*LR Chi-square (P-value)*		131.51 *(P < 0.001)*		550.95 *(P < 0.001)*		303.15 (*P* = 0.005)
*Linktest hat-OR (P-value)*		2.63 *(P < 0.001)*		3.98 *(P < 0.001)*		2.62 (P < 0.001)
*Hosmer-Lemeshow statistic* *(P-value)*		15.98 *(P < 0.001)*		11.78 *(P = 0.001)*		14.54 (*P* = 0.002)
*Area under ROC curve*		0.79		0.72		0.75
*VIF Mean (Max)*		3.21 (4.56)		2.45 (3.96)		2.90 (4.13)
Participants characteristics	European		Overall			
	Un-adjusted OR (95% CI)	Adjusted OR (95% CI)	Un-adjusted OR (95% CI)		Adjusted OR (95% CI)	
Age group						
40–44 years (= ref)	1.00	–	1.00		1.00	
≥ 45 years	1.23 (0.75, 2.03)		0.86 (0.84, 0.89)		0.91 (0.87, 0.94)	
Educational level						
*No education (= ref)*	1.00	1.00	1.00		1.00	
*Primary*	0.95 (0.56, 1.97)	4.01 (3.63, 4.43)	2.44 (2.32, 2.56)		2.39 (2.25, 2.53)	
*Secondary*	1.97 (1.56, 2.49)	3.55 (3.20, 3.94)	4.01 (3.82, 4.20)		2.08 (1.95, 2.21)	
*Higher*	1.57 (1.39, 1.76)	3.24 (2.83, 3.73)	6.50 (6.16, 6.86)		2.48 (2.25, 2.73)	
Gender of the household head						
*Male (= ref)*	1.00	–	1.00		1.00	
*Female*	1.40 (0.29, 6.69)		1.19 (1.15, 1.23)		1.65 (1.58, 1.73)	
Respondent’s age at 1st birth						
*< 18 years (= ref)*	1.00	–	1.00		1.00	
*18–20 years*	2.57 (0.24, 7.29)		1.51 (1.45, 1.58)		1.19 (1.13, 1.26)	
*21–25 years*	2.86 (0.28, 8.92)		1.93 (1.85, 2.02)		1.30 (1.23, 1.38)	
*> 25 years*	1.35 (0.32, 4.81)		2.40 (2.27, 2.53)		1.65 (1.52, 1.78)	
Number of childbirths						
*< 4 (= ref)*	1.00	–	1.00		1.00	
*4–5*	0.59 (0.35, 1.00)		1.11 (1.07, 1.15)		1.25 (1.18, 1.31)	
*> 5*	0.33 (0.10, 1.02)		1.21 (1.17, 1.26)		1.48 (1.40, 1.57)	
Mass media exposure						
*No (= ref)*	1.00	1.00	1.00		1.00	
*Yes*	6.02 (1.54, 23.59)	1.86 (1.79, 1.93)	1.93 (1.85, 2.02)		1.84 (1.79, 1.89)	
Health insurance coverage						
*No (= ref)*	1.00	1.00	1.00		1.00	
*Yes*	3.19 (1.65, 6.19)	2.60 (2.42, 2.80)	1.32 (1.27, 1.38)		1.09 (1.04, 1.14)	
Body mass index						
*Under weight*	ns		0.68 (0.62, 0.75)		0.96 (0.87, 1.06)	
*Normal weight (= ref)*	1.00	–	1.00		1.00	
*Overweight*	1.49 (0.92, 2.43)		1.43 (1.37, 1.49)		1.04 (0.98, 1.09)	
*Obese*	1.27 (0.70, 2.32)		1.44 (1.38, 1.51)		0.89 (0.84, 0.94)	
Community						
*Urban*	2.88 (1.75, 4.75)	1.13 (1.05, 1.22)	2.19 (2.12, 2.25)		1.20 (1.14, 1.26)	
*Rural (= ref)*	1.00	1.00	1.00		1.00	
Wealth quintile						
*Q1 (Poorest 20%) (= ref)*	1.00	1.00	1.00		1.00	
*Q2*	1.07 (0.59, 1.94)	1.44 (1.30, 1.60)	1.08 (1.03, 1.14)		1.26 (1.17, 1.36)	
*Q3*	1.96 (1.06, 3.62)	1.46 (1.32, 1.63)	1.21 (1.15, 1.27)		1.17 (1.08, 1.26)	
*Q4*	3.40 (1.63, 7.07)	1.65 (1.47, 1.84)	1.46 (1.39, 1.53)		1.43 (1.31, 1.55)	
*Q5 (Richest 20%)*	1.27 (1.08, 3.17)	2.05 (1.81, 2.32)	2.10 (2.00, 2.20)		2.01 (1.84, 2.20)	
*LR Chi-square (P-value)*		130.46 (*P* < 0.005)			375.12 (P < 0.001)	
*Linktest hat-OR (P-value)*		2.98 (P < 0.001)			3.26 (P < 0.005)	
*Hosmer-Lemeshow statistic (P-value)*		75.19 (*P* < 0.002)			20.62	
*Area under ROC curve*		0.80			0.80	
*VIF Mean (Max)*		3.16 (3.15)			3.20 (3.51)	

Participants living in households with access to mass media communication were significantly (1.84 times higher) users of BCS services (OR = 1.84, 95% CI: 1.79–1.89) compared with households that did not have exposure to mass media communication. Households with health insurance coverage showed a 1.09 times (OR = 1.09, 95% CI: 1.04–1.14) higher use of BCS services compared to households without health insurance coverage. Richest and moderate economic situation were associated with 2.01 times (OR = 2.01, 95% CI: 1.84–2.20) and 1.43 times (OR = 1.43, 95% CI: 1.31–1.55) higher use of BCS services, compared to poorest households. Additionally, the study found that the use of BCS services among obese participants was 11% (OR = 0.89, 95% CI: 0.84–0.94) lower compared to their healthy weight peers after controlling other factors. Furthermore, women who lived in urban communities used more screening services (OR = 1.20, 95% CI: 1.14–1.26) compared with women who lived in rural communities.

## Discussion

The results show that the overall utilisation of BCS services in the 14 LRCs was 15.41% (95% CI: 15.22–15.60%), varying from 81.10% (95% CI: 76.85 to 84.73%) in the European country, 18.61% (95% CI: 18.16 to 19.06%) in Asian countries, 14.30% (95% CI: 13.61 to 14.96%) in American countries, and 14.29% (95% CI: 13.87 to 14.74%) in African countries. The utilisation of BCS services varied across countries and geographical areas, influenced by social and cultural norms, religious beliefs, health knowledge, and awareness. Other factors influence the use of BCS services, although the current study focused only on predisposing factors, enabling factors, economic status, and body mass index as the predictors of BCS services. The findings exhibit that the factors that significantly contributed to the likelihood of using BCS services included higher levels of educational background and participants being from a female-headed household.

The results indicate that a higher level of education significantly associated women’s uptake of breast cancer screening services. This finding is in line with a recent research finding, in which higher educated women were significantly associated with higher utilisation of BCS services compared to participants with lower levels of educational background [23]. Higher edicated women are more aware of health complications and adverse effects of diseases, including reproductive health check-up, screening services (e.g., breast cancer, cervical cancer), and prevention strategies (e.g., screening services, vaccinations), and thus more likely to use the BCS services. Therefore, interventions to increase the participation rates for BCS services may emphasis specifically on those with lower education levels or may focus on increasing women’s health education and awareness levels to achieve population-level increments in screening services.

The present study also revealed that participant’s household access to mass media coverage were significantly correlated with more utilisation of BCS services. A previous research has found that media exposure was generally cited as the primary vehicles for increasing awareness about breast cancer screening services and early detection strategies, including breast screening or breast examination associated services [22]. This contributes toward improving overall awareness when implementing new interventions correlated to health programs for primary breast cancer detection [62, 63]. Other studies have found that mass media communications were significantly associated with the higher utilisation of cancer screening services [64–68]. Some interventions based on media communication to increase the use of women cancer screening services and screening services have been revealed to improve screening behaviour of women by nearly 4 to 10% [66, 67]. Therefore, governments should deliberate initiatives that produce program awareness about breast cancer screening services through extensive broadcast associated health messages, or through modes most likely to be promoted in each country context or geographical lens.

The findings show that women’s BMI status was significantly associated with the utilisation of BCS services. In a prior study [24], the researchers also revealed that obese or overweight women utilised 17% less screening services in comparison with women of healthy weight. However, the risk of BC was higher in amongst obese women (> 35 kg/m^2^) [69, 70]. Among older women, obese (≥ 30 kg/m^2^) women were associated with higher risk of occurring BC, compared to healthy women, while that relationship tended to be the inverse in reproductive women [51]. More research (e.g., quantitative exploration including randomised control trials, clinical trials, and epidemiological studies) is significantly to investigate the reasons for this lower utilisation so that efforts can be made to promote BCS rates.

The results further showed that women with health insurance coverage were significantly associated with higher utilisation of BCS services compared to their uninsured counterparts. This finding is consistent with the previous finding [71], in which it was found that women from insured households had a 70% higher utilisation of BCS. Here, the possible reason could be a high out-of-pocket payment that hinders access. Previous research has provided evidence that low incomes, being uninsured, and lack of affordability of healthcare services were significantly correlated with a lower probability of utilising BCS services [71–77]. Thus, the current results provide further evidence in relation to health care systems that do not incorporate community-based healthcare programs. These programs, including chronic disease management, health promotion, and awareness, affordable services, etc., and those who do not make BCS services available as part of existing healthcare packages, are likely to experience lower than optimal screening rates. This, in turn, will lead to a higher breast cancer burden and lower survival rates. Hence, it is necessary to address the financial barriers associated with BCS services amongst the uninsured in low resource countries.

The findings also identified socio-economic status as another significant predictor that makes the richest women more likely to undergo BCS services compared to the poorest women. The conclusion that the participants with the wealthiest economic conditions are significantly more likely the use screening services in low-resource countries aligns with previous results [78–80]. The results further showed that urban residence leads to higher use of BCS services, which is also support with previous research [81]. By contrast, another study has revealed that the use of screening services was comparatively high among women living in rural areas [11]. The most common reasons for women in urban communities being more likely to use the BCS services considering affordability, accessibility, and availability of services [82–87]. Therefore, accessible screening facilities may increase the use of screening services. Mobile-based screening for cancer should be increased in low-resource settings to target rural area womens’ BCS uptake. Other interventions in low-resource settings could include community health workers guided by smartphone applications. This model can play an active and significant role in confirming breast health promotion, which contributes to increasing participation in screening services [87–93].

This study has some limitations. The data were derived from the latest DHS, which is based on self-reported information of respondents. The present study finindgs were derived based on self-reported data that might occur recall and social desirability bias. As a result, there might be a risk that screening-related estimates were over-reported. Further studies might confirm these results. Furthermore, this study was cross-sectional in design; hence it can not provide an exploration of causal inferences. Some common quantitative factors that have been used in similar prior studies, such as marital status, past screening behaviors, previous knowledge on screening, religion, cultural beliefs, provider attitude, the side effect of the screening, demand for healthcare, and costs of screening services, were excluded due to the lack of data in the DHS survey. These types of factors should consider in the further study in terms of the health system and societal perspectives, which might significant for policymaker or researchers to develop an appropriate program design or intervention (e.g., patient preferences) to reduce the burden of breast cancer among high risk or disadvantaged communities. Another limitation is that the participants’ binary responses did not allow cross-validation of qualitative data. Also, the questions related to the use of BCS services varies across countries, and they depend on country-specific cultural beliefs and social norms. Although, the DHS data are nationally representative and countywide among reproductive women. The present study was a sub-study. The present study participates were women aged 40 or above due to the high risk of breast cancer incidence at this age group. However, the breast screening facility might be localised on region or city of the country.

In this context, the present study constructed based on 14 LRCs where breast cancer screening services-related variables were available. The present study has produced to pooled findings, which incorporate a more precise estimate across possible geographical areas. In the context of the European region, data associated with BCS services are available for only a single country (e.g., Albania). The prevalence and association of BCS services may therefore underestimate or overestimate for this region. Further investigations are necessary to confirm more precise estimates, including additional countries and settings in the European region. The authors have reviewed questions related to breast cancer screening services in the DHS datasets and identified the different forms of questions across the study countries. The dependent variable (i.e., the ever use of BCS practice) was restricted to DHS data based on the various types of items related to breast cancer screening services, which might create a concern about whether the outcomes are combined in an ‘overall’ estimate.

Despite these limitations, the strength of the study is that it has used nationally representative data that have been gathered following standardised scientific procedures, and public health researchers broadly use these data. The main strength of the present study was the large sample size, which included 140,974 reproductive women in 14 LRCs across international geographical lens. This large sample size may offer more precise estimates as limited settings, or small-scale studies, are only able to draw on a small piece of evidence related to screening services in a regional or community’s context. Further, the inclusion of predictors beyond simple demographics is another strength. Finally, this study included new factors such as media exposure and nutritional status to check if any associations existed with the usage of BCS services.

## Conclusions

Breast cancer screening (BCS) services are very important for LRCs where the burden of BC is generally poorly documented, while its impact on the population is large and growing. To combat this burden, its magnitude must be outlined so that regular screening services for the early detection of BC can be planned efficiently by standard healthcare facilities, so that prevention mechanisms can be improved. Despite the benefits of BCS services, the utilisation of these services is very low in LRCs, although this varies widely from country to country. The findings show that education, age at first birth, head of household status, mass media communication health insurance coverage, economic status, nutritional status, and rural residence have a positive influence on higher use of BCS services. However, the magnitude of association varies across countries, with wide cultural diversity in the studied countries. The findings emphasize that culturally appropriate promotional campaigns, health awarness, health education programs, and health policy, aimed at these socio-economically and geographically disadvantaged women, might be helpful when promoting BCS services.

A better understanding of public healthcare systems, with regards to access to advanced medical technologies or BCS services may help to understand the variations in screening observed. The utilisation of BCS services is advantageous when it is performed in a structured and regular manner in well run public health systems or when the per capita income of the population permits individuals to absorb most of the associated expenses. Country-specific qualitative studies are required to explore the main reasons, challenges, and barriers for the lower use of BCS services in a cross-cultural context.

## Data Availability

The DHS data are publicly accessible by the Measure DHS. https://dhsprogram.com/data/

## Abbreviations

BCS: Breast cancer screening
VIF: Variance Influential Factors
OR: Odds Ratios
CI: Confidence Interval
DHS: Demographic and Health Survey
LSR: Low-resource country
